# Optimization of ISAC Trade-Off via Covariance Matrix Allocation in Multi-User Systems

**DOI:** 10.3390/e27111144

**Published:** 2025-11-09

**Authors:** Danaisy Prado-Alvarez, Daniel Calabuig, Saúl Inca, Jose F. Monserrat

**Affiliations:** iTEAM Research Institute, Universitat Politècnica de València, 46022 Valencia, Spain; dacaso@iteam.upv.es (D.C.); sauin@iteam.upv.es (S.I.); jomondel@iteam.upv.es (J.F.M.)

**Keywords:** ISAC, trade-off analysis, multi-user MIMO, resource optimization, condition number

## Abstract

Integrated Sensing and Communication (ISAC) is envisioned as a foundational technology for future wireless networks, enabling simultaneous wireless communication and environmental sensing using shared resources. A key challenge in ISAC systems lies in managing the trade-off between communication data rate and sensing accuracy, especially in multi-user scenarios. In this work, we investigate the joint design of transmit signal covariance matrices to optimize the sum data rate while ensuring certain sensing performance. Specifically, we formulate a constrained optimization problem where the transmit covariance matrix is allocated to maximize the communication sum-rate under sensing-related constraints. These constraints condition the design of the transmit signal’s covariance matrix, impacting both the sensing channel estimation error and the sum data rate. Our proposed method leverages convex optimization tools to achieve a principled balance between communication and sensing. Numerical results demonstrate that the proposed approach effectively manages the ISAC trade-off, achieving near-optimal communication performance while satisfying sensing requirements.

## 1. Introduction

Integrated Sensing and Communication (ISAC) has emerged as a key paradigm for next-generation wireless networks, where a common hardware platform and joint signal processing are leveraged to simultaneously support communication and sensing functionalities [1,2]. By enabling spectrum sharing and hardware reuse, ISAC offers significant advantages regarding spectral efficiency, energy consumption, and system cost, which are critical for future applications such as autonomous driving, smart cities, and industrial automation.

Despite its potential, one of the fundamental challenges in ISAC systems lies in managing the inherent trade-off between communication performance and sensing accuracy. Due to the shared use of resources, such as transmit signal, power, and spatial degrees of freedom (DoF), optimizing the system for communication often degrades the sensing performance, and vice versa. Thus, effective ISAC design necessitates a careful balance between these competing objectives [1].

This trade-off is most evident in the design of transmit signal structures, where communication and sensing often require conflicting properties. Communication schemes aim to maximize the data rate or reliability, favoring directional beamforming and power allocation strategies aligned with user channel eigenmodes. In contrast, sensing tasks such as target detection or parameter estimation benefit from broader spatial coverage, diversified or orthogonal signal structures, and more uniform energy allocation to enhance resolution and robustness. These differences are fundamentally reflected in the transmit covariance matrix, which governs the spatial and spectral properties of the transmitted signal. Although communication-oriented designs optimize the covariance matrix for spectral efficiency, often through water filling or eigenmode transmission, sensing-centric designs instead emphasize uniform or structured energy distribution, with performance typically assessed through criteria such as Cramér-Rao bound (CRB) or mean square error (MSE) [2,3].

One straightforward approach to address this trade-off is to transmit in alternating time slots. In some intervals, the system employs the transmit covariance matrix optimized for communication, while in others, it uses the one optimized for sensing. This strategy, known as time-sharing [4], allows for a balanced overall performance between the two functionalities. However, as demonstrated in [4], there is a gap between the performance of simple time-sharing schemes and the Pareto frontier, indicating potential for improvement. In addition, when the sensing-optimal matrix is applied, communication continues but with degraded performance. Similarly, when the communication-optimal matrix is used, sensing persists, although its quality may deteriorate. These limitations render time-sharing unsuitable for dynamic scenarios such as unmanned aerial vehicle (UAV)-enabled ISAC systems [5], where continuous sensing or maintaining a minimum sensing quality is essential.

Another approach is to combine the communication and sensing objectives into a single scalar function using a weighted sum [6]. This allows both objectives to be optimized jointly, rather than alternated as in time-sharing, which improves the overall trade-off between communication and sensing. The performance of this method depends on the chosen weights, which balance the system according to predefined priorities. However, weighted-sum optimization does not guarantee a minimum performance for either function. To address this limitation, a constraint can be imposed on one of the objectives while optimizing the other, providing explicit control over the guaranteed performance level of the constrained task [7,8].

In our work, we adopt the latter approach, as it allows us to enforce a desired sensing requirement while optimizing communication performance. Specifically, we maximize communication under sensing-aware constraints. We analyze the sensing and communication trade-off under different antenna configurations, including multi-user scenarios where the number of transmit antennas may be greater than, equal to, or fewer than the sum of receive antennas across all users. In these cases, the number of channel DoF determines how many independent communication streams can be transmitted and how many spatial directions can be effectively sensed simultaneously. Systems with more DoF can better support both high communication rates and accurate sensing, while systems with fewer DoF require a careful trade-off between the two objectives. These results provide practical guidance for designing ISAC systems and illustrate the effectiveness of our framework across a variety of deployment scenarios.

## 2. System Model

We consider an ISAC system with a transmitter equipped with $N_{t}$ antennas, a sensing receiver equipped with $N_{r}$ antennas, and *K* user equipments (UEs), each equipped with $N_{u}$ antennas.

The transmitted signals are denoted by $X_{k}\in C^{N_{t}\times L}$ for k=0,1,…,K, where $X_{0}$ is a dedicated sensing signal and *L* is the number of Orthogonal Frequency Division Multiplexing (OFDM) symbols. The covariance matrix of each column of these signals is $Q_{k}=E\left[X_{k}\left(:,n\right)X_{k}{\left(:,n\right)}^{*}\right]$ for k=0,…,K and n=1,…,L.

The communication channel from the transmitter to the *k*-th UE is denoted by $H_{k}\in C^{N_{u}\times N_{t}}$, k=1,…,K. The received signal at the *k*-th user is

(1) $$Y_{k}=H_{k}\left(\sum_{k=0}^{K}X_{k}\right)+Z_{k},\;Y_{k},Z_{k}\in C^{N_{u}\times L},$$

where $Z_{k}$ represents additive noise with i.i.d. entries $Z_{k}∼\mathrm{CN}\left(0,\sigma_{c}\right)$. For convenience, we define ${\bar{H}}_{k}=H_{k}/\sigma_{c}$.

The sensing channel is denoted by $H_{0}\in C^{N_{r}\times N_{t}}$, and the received signal at the sensing receiver is

(2) $$Y_{0}=H_{0}\left(\sum_{k=0}^{K}X_{k}\right)+Z_{0},\;Y_{0},Z_{0}\in C^{N_{r}\times L},$$

with $Z_{0}∼\mathrm{CN}\left(0,\sigma_{s}\right)$ and ${\bar{H}}_{0}=H_{0}/\sigma_{s}$.

For communications, the transmitter employs dirty paper coding (DPC) [9] to encode user signals due to its capacity-achieving property in multi-user Multiple-Input Multiple-Output (MIMO). In DPC, users are encoded sequentially, allowing the transmitter to pre-cancel interference from previously encoded users by treating it as known. Consequently, each user only experiences interference from those encoded later, with the last user in the sequence enjoying an interference-free channel. The achievable rate for user *i* under DPC is

(3) $$R_{i}\left({\left(Q_{k}\right)}_{k=1}^{K}\right)=\log\frac{\left|I+\bar{H}i\left(\sum k\geq iQ_{k}\right)\bar{H}i^{*}\right|}{\left|I+\bar{H}i\left(\sum k>iQ_{k}\right)\bar{H}i^{*}\right|},$$

where the encoding order k=1,…,K determines the interference structure. The numerator includes the user’s signal and interference from later users, while the denominator excludes the user’s contribution, isolating the remaining interference. This structure enables DPC to achieve the Gaussian broadcast channel (BC) capacity under a sum-power constraint. The sensing signal $X_{0}$, known at the base station (BS), does not interfere with the DPC chain, affecting only the total available transmit power.

For sensing, the maximum likelihood (ML) estimator [3] is used to estimate $H_{0}$:

(4) $${\hat{H}}_{0}=Y_{0}X^{*}{\left(XX^{*}\right)}^{-1},\;\mathrm{where}\;X=\sum_{k=0}^{K}X_{k}.$$

The ML estimator is optimal under Gaussian noise and known channel statistics. Its performance depends heavily on the conditioning of the matrix $XX^{*}$. As the number of OFDM symbols *L* grows, the expectation of $XX^{*}$ converges to $L\sum_{k=0}^{K}Q_{k}$. Therefore, ensuring that $\sum_{k=0}^{K}Q_{k}$ is well-conditioned improves the accuracy of the channel estimate. The sensing performance is quantified by the normalized mean square error (NMSE):

(5) $$\mathrm{NMSE}=\frac{∥H_{0}-{\hat{H}}_{0}{∥}_{F}^{2}}{∥H_{0}{∥}_{F}^{2}}.$$

It is important to note that our goal is not to address a specific sensing application. Instead, we focus on the underlying channel estimation process, which serves as the fundamental enabler of all sensing functionalities. Accurate channel knowledge determines the performance limits of downstream tasks. By assessing the NMSE of the estimated sensing channel, we capture a core, application-agnostic measure of sensing performance, complementary to the sum data rate for communication.

## 3. Problem Statement

The design of the covariance matrices $Q_{k}$ is critical for balancing the ISAC trade-off. On one hand, they determine the power allocation and spatial transmission strategy for communication; conversely, they affect the invertibility and conditioning of the effective sensing matrix, thus influencing estimation accuracy.

We formulate the following optimization problem to maximize the communication sum-rate subject to sensing and power constraints:

(6) $$\begin{array}{cc} \max_{{\left(Q_{k}\right)}_{k=0}^{K}} & \;\sum_{i=1}^{K}R_{i}\left({\left(Q_{k}\right)}_{k=0}^{K}\right)\\ s.t.\; & Q_{k}⪰0\;\forall k=0,\ldots ,K,\\ \; & \mathrm{Tr}\left(\sum_{k=0}^{K}Q_{k}\right)\leq P_{T},\\ \; & \mathrm{Cond}\left(\sum_{k=0}^{K}Q_{k}\right)\leq \alpha ,\\ \; & \mathrm{Tr}\left(Q_{0}\right)\geq P_{S}. \end{array}$$

There are four constraints in this problem:

1. Positive Semi-Definiteness of covariance matrices: Each matrix $Q_{k}$ must be positive semi-definite, meaning that all eigenvalues of $Q_{k}$ must be non-negative. This statement is a standard constraint in optimization involving matrices, especially when they represent covariance or power matrices.
2. Total power constraint: The trace of the sum of all matrices $Q_{k}$ must be less than or equal to a specified threshold $P_{T}$. The matrix trace represents the total available power, so this constraint limits the total power budget available across all matrices $Q_{k}$.
3. Condition number constraint: The condition number of the sum $\sum_{k=0}^{K}Q_{k}$ is constrained to be no greater than α. The condition number measures the sensitivity of the matrix to inversion or linear stability, and limiting it helps control the numerical properties of the matrix sum. This is relevant for the sensing channel estimation, where it is convenient to guarantee the invertibility of $\sum_{k=0}^{K}Q_{k}$.
4. Threshold power for sensing: The trace of $Q_{0}$ must be at least $P_{S}$, imposing a minimum power specifically on $Q_{0}$. This guarantees a minimum allocation of power to sensing.

This formulation enables the joint optimization of communication and sensing resources, embedding sensing quality directly into the communication design via tractable constraints.

## 4. Use Cases

Having outlined the general problem, we now focus on three specific cases that address key questions of interest. First, we consider what would be optimal for communications. Next, we examine whether it is possible to ensure a certain level of sensing performance without a dedicated sensing signal and without significantly impacting communications. Finally, we explore how having a dedicated sensing signal benefits sensing and affects communications. Below, we present the three cases in detail.

### 4.1. The Best for Communication

The optimal solution for communications can be achieved by assuming, within the general problem, that α=∞ and $P_{S}=0$. In other words, there are no constraints on the condition number, and all available power is allocated solely to communications. Given these assumptions, the general problem simplifies to an equivalent problem, which can be expressed as follows:

(7) $$\begin{array}{cc} \max_{{\left(Q_{k}\right)}_{k=1}^{K}} & \;\sum_{i=1}^{K}R_{i}\left({\left(Q_{k}\right)}_{k=1}^{K}\right)\\ s.t. & \;Q_{k}⪰0\;\mathrm{for}\;k=1,\ldots ,K,\\ \; & \;\mathrm{Tr}\left(\sum_{k=1}^{K}Q_{k}\right)\leq P_{T}. \end{array}$$

The sum-rate maximization problem in the broadcast channel is non-convex. This non-convexity arises from the presence of inter-user interference, which results in each user’s rate being expressed as the logarithm of a matrix fraction (signal over interference-plus-noise). Therefore, the sum of these log-fractions cannot be directly formulated as a convex problem. In our formulation, we adopt the standard transformation to the dual multiple access channel (MAC) domain [10], where the sum-rate expression becomes concave in the covariance matrices, allowing us to efficiently solve the communication optimization problem using interior-point methods and then map power allocations and rate regions from the MAC back to the BC as explained in [11]. This step corresponds to a well-established convex reformulation in MIMO broadcast optimization.

While solving this problem achieves maximum communication capacity, there are no constraints to guarantee a minimum performance level for sensing functionalities. To address this, we propose two more alternative approaches.

### 4.2. Guaranteeing Specific Sensing Performance Without a Sensing Dedicated Signal

This approach ensures that $\sum_{k}Q_{k}$ is a well-conditioned matrix, even without a dedicated sensing signal. This condition improves the stability and accuracy of sensing by indirectly supporting channel estimation through the structure of the communication covariance matrices. The equivalent problem can be written as follows:

(8) $$\begin{array}{cc} \max_{{\left(Q_{k}\right)}_{k=1}^{K}} & \;\sum_{i=1}^{K}R_{i}\left({\left(Q_{k}\right)}_{k=1}^{K}\right)\\ s.t. & \;Q_{k}⪰0\;\mathrm{for}\;k=1,\ldots ,K,\\ \; & \;\mathrm{Tr}\left(\sum_{k=1}^{K}Q_{k}\right)\leq P_{T},\\ \; & \;\mathrm{Cond}\left(\sum_{k=1}^{K}Q_{k}\right)\leq \alpha . \end{array}$$

A well-conditioned covariance matrix, with a condition number below α, ensures that the matrix is close to invertible and has balanced eigenvalues. This balance is crucial for the estimator, as it relies on inversions of such matrices to accurately estimate the sensing channel. When $\sum_{k}Q_{k}$ is well-conditioned, it minimizes noise amplification and errors during the estimation process, leading to a more precise sensing channel estimate.

Typically, optimization problems in BC can be transformed to their dual in the MAC to simplify solving, as previously done. However, introducing a condition number constraint on $\sum_{k}Q_{k}$ makes this approach unfeasible. The condition number constraint is not preserved in the dual transformation because the condition of $\sum_{k}Q_{k}$ directly affects the covariance matrix in the original BC formulation. In the dual MAC, such conditioning constraints cannot be directly mapped, making it impossible to achieve an equivalent dual problem that respects the well-conditioning requirement. Thus, the dual approach is unsuitable with this constraint.

To overcome this limitation, we develop a bisection-based algorithm specifically designed for conditioning control in the transmit covariance structure. This algorithm enables practical enforcement of the desired condition number while maintaining near-optimal communication performance. In essence, it allows the system to systematically balance the total transmit power and the eigenvalue distribution of the aggregate covariance matrix, ensuring that the resulting transmission remains well-conditioned without sacrificing data rate efficiency. By iteratively refining these parameters, the algorithm effectively bridges the gap between theoretical optimization and implementable system design under structural constraints.

As a first step, the initial covariance matrices $Q_{k}$ are obtained by solving the original communication optimization problem without imposing any condition number constraint, thus providing a performance-oriented but potentially ill-conditioned starting point. The algorithm first checks whether this initial allocation already satisfies the desired condition-number threshold. If so, it is directly accepted. Otherwise, a bisection-based iterative process is initiated to jointly adjust the total transmit power and the eigenvalue distribution of the aggregate covariance $\sum_{k}Q_{k}$. At each iteration, the communication optimization problem is solved in the MAC domain under the current trial power budget. The resulting sum covariance is then checked against the condition-number threshold α. If the constraint is violated, the eigenvalues of the sum covariance are capped to enforce the desired condition number, producing a feasible covariance projection. The power bounds are then updated according to the feasibility outcome, guiding the next iteration of the bisection search.

Following eigenvalue capping, the algorithm computes the correction matrix, which quantifies the excess power along each eigen-direction required to satisfy the condition-number constraint. This correction is added entirely to the covariance of the first user in the encoding order. By doing so, the method preserves the spatial structure of the aggregate covariance, as the excess power is redistributed according to the original eigen-directions rather than isotropically. Assigning the correction to the first user minimizes the impact on the achievable sum-rate of the other users, since this user inherently experiences interference from all following users. Through this iterative bisection procedure, the algorithm continuously updates the power bounds to approach the maximum feasible transmit power while guaranteeing that the condition-number constraint is satisfied, maintaining both communication performance and numerical stability.

The procedure is detailed in Algorithm 1.
**Algorithm 1** Bisection-Based Algorithm for Conditioning Control 1:**Input:** Initial feasible covariances $\left\{Q_{k}\right\}$, condition-number threshold α, total power budget $P_{T}$, tolerance ϵ 2:**Output:** Adjusted covariances $\left\{Q_{k}^{\mathrm{opt}}\right\}$ 3:Compute initial sum covariance: $S_{\mathrm{init}}=\sum_{k=1}^{K}Q_{k}$ 4:**if** condition number of $S_{\mathrm{init}}\leq \alpha$ **then** 5:       Accept initial allocation: $\left\{Q_{k}^{\mathrm{opt}}\right\}=\left\{Q_{k}\right\}$ 6:**end if** 7:Initialize bisection bounds: $P_{\min}=0$, $P_{\max}=P_{T}$ 8:**while** $P_{\max}-P_{\min}>\epsilon$ **do** 9:       Set current total power: $P_{\mathrm{current}}=\left(P_{\min}+P_{\max}\right)/2$10:      Solve the convex communication optimization in MAC domain with total power $P_{\mathrm{current}}$ to obtain $\left\{Q_{k}\right\}$11:      Compute sum covariance: $S=\sum_{k}Q_{k}$12:      **if** condition number of S≤α **then**13:            Update best feasible allocation: $\left\{Q_{k}^{\mathrm{opt}}\right\}=\left\{Q_{k}\right\}$14:            Update lower bound: $P_{\min}=P_{\mathrm{current}}$15:      **else**16:            Eigen-decompose sum covariance: $S=V\Lambda V^{†}$, $\Lambda =\mathrm{diag}(\lambda_{1},\ldots ,\lambda_{N_{t}})$17:            Cap eigenvalues: $\lambda_{i}^{\prime }=\min\left(\lambda_{i},\alpha \cdot \lambda_{\min}\right)$ for all *i*18:            Form capped covariance: $S^{\prime }=V\;\mathrm{diag}\left(\lambda_{1}^{\prime },\ldots ,\lambda_{N_{t}}^{\prime }\right)\;V^{†}$19:            Compute correction matrix: $\Delta =S-S^{\prime }$20:            Add correction entirely to first user’s covariance: $Q_{1}^{\mathrm{new}}=Q_{1}+\Delta$21:            Update upper bound: $P_{\max}=P_{\mathrm{current}}$22:      **end if**23:**end while**24:**return** $\left\{Q_{k}^{\mathrm{opt}}\right\}$ as the adjusted covariances

The bisection converges deterministically in $\log_{2}\left(\frac{P_{\max}-P_{\min}}{\epsilon }\right)$ iterations. Each iteration solves a convex sum-rate maximization problem with dominant complexity $O(K^{4}N_{t}^{7})$, while eigen-decomposition and eigenvalue adjustment have lower-order polynomial cost $O(N_{t}^{3})$.

### 4.3. Guaranteeing Specific Sensing Performance Using a Sensing Dedicated Signal

This approach allocates a portion of the total power specifically for sensing, improving sensing performance by enhancing channel estimation accuracy. The simplified problem can be written as:

(9) $$\begin{array}{cc} \max_{{\left(Q_{k}\right)}_{k=0}^{K}} & \;\sum_{i=1}^{K}R_{i}\left({\left(Q_{k}\right)}_{k=0}^{K}\right)\\ s.t. & \;Q_{k}⪰0\;\mathrm{for}\;k=0,\ldots ,K,\\ \; & \;\mathrm{Tr}\left(\sum_{k=0}^{K}Q_{k}\right)\leq P_{T},\\ \; & \;\mathrm{Tr}\left(Q_{0}\right)\geq P_{S}, \end{array}$$

where $Q_{0}$ is designed as a scaled identity matrix, optimal for channel estimation [12]. With a dedicated sensing signal $X_{0}$ and its corresponding covariance matrix $Q_{0}$, the sum of the covariance matrices $\sum_{k=0}^{K}Q_{k}$ inherently satisfies a bounded condition number. Since $Q_{0}$ is chosen as a scaled identity matrix with trace $P_{S}$, each of its eigenvalues equals $\frac{P_{S}}{N_{t}}$, which guarantees that the minimum eigenvalue of the overall covariance matrix is at least $\frac{P_{S}}{N_{t}}$. In the worst-case for conditioning, the remaining communication power $P_{T}-P_{S}$ is concentrated in a single eigen-direction. Therefore, in the worst case, the condition number can be expressed as

(10) $$\alpha =N_{t}\left(\frac{P_{T}}{P_{S}}-1\right)+1.$$

The explicit condition number constraint can be omitted because the sensing power allocation automatically enforces this upper bound.

It was previously mentioned that having a dedicated sensing signal does not impact the communication rate calculation beyond the power allocated for sensing, due to the use of DPC. With this in mind, the optimization problem can be reformulated as an equivalent problem where the total power $P_{T}$ is reduced by the sensing power $P_{S}$.

(11) $$\begin{array}{cc} \max_{{\left(Q_{k}\right)}_{k=1}^{K}} & \;\sum_{i=1}^{K}R_{i}\left({\left(Q_{k}\right)}_{k=1}^{K}\right)\\ s.t. & \;Q_{k}⪰0\;\mathrm{for}\;k=1,\ldots ,K,\\ \; & \;\mathrm{Tr}\left(\sum_{k=1}^{K}Q_{k}\right)\leq P_{T}-P_{S}. \end{array}$$

Subsequently, the problem is solved as problem (7).

## 5. Evaluation Results

To estimate a channel, it is necessary to probe all orthogonal directions defined by its singular vectors. Under the assumption $N_{r}=N_{t}$, which is typical for monostatic sensing where the same node transmits and receives the signal, a sensing channel of dimension $N_{r}\times N_{t}$ has $N_{t}$ singular vectors, corresponding to $N_{t}$ orthogonal directions or DoF. In contrast, a communication channel of dimension $N_{u}\times N_{t}$, with $N_{u}<N_{t}$, effectively supports only $N_{u}$ DoF. To maximize data rate, transmit power should be concentrated on these $N_{u}$ dominant directions, rather than being spread across the unused $N_{t}-N_{u}$ directions. Balancing this trade-off is critical, as leveraging the full $N_{t}$ DoF is essential for accurate sensing, but only $N_{u}$ DoF contribute directly to communication. This tension forms the basis for the analysis presented in this section.

This evaluation considers a urban macro-cell (UMa) scenario, featuring a single BS and one sector site or cell with a 500-m diameter. The BS is centrally positioned in the cell at a height of 25 m. Communication users (or UEs) are randomly distributed within this area at a height of 1.5 m, with a minimum 3D distance of 1.5 m from the BS.

For sensing, a mono-static mode is implemented, where the BS both transmits and receives the signal used for sensing. The target for sensing is randomly positioned within the environment and always maintains a line of sight (LoS) condition relative to the BS. Communication users’ LoS or non line of sight (NLoS) conditions follow the Third Generation Partnership Project (3GPP) model specifications [13], which determine visibility based on urban propagation factors.

The BS has a 32×1 antenna array, while each UE has a linear 4×1 array. All antenna elements are spaced λ/2 apart, where λ represents the signal wavelength.

Table 1 provides the detailed configuration parameters for the evaluated scenario. Building on the network layout described previously, the evaluation requires a large set of random seeds, approximately 10,000, to ensure robust statistical reliability. The number of OFDM symbols (*L*) for channel estimation varies from 64 to 2048.

Following guidelines from Table 7.8-1 in TR 38.901 [13], certain simulation assumptions are applied for the communication-only scenarios, including parameters like carrier frequency, sampling frequency, and the number of subcarriers. The hybrid ISAC channel model from [14] is utilized for sensing functions, while the 3GPP channel model supports the communication aspects. In our setup, the communication and sensing channels may exhibit some correlation due to the shared environment, although such correlation is not required for the evaluation performed in this study. This setup enables a comprehensive assessment of the performance trade-offs in the ISAC system under realistic conditions.

In this study, an underdetermined system refers to scenarios where $N_{t}>N_{u}$. Such systems are flexible but underutilized, leaving resources untapped. In contrast, an overdetermined system arises when $N_{t}\leq N_{u}$. These systems are resource-constrained but better conditioned for optimal performance.

**Figure 1:**
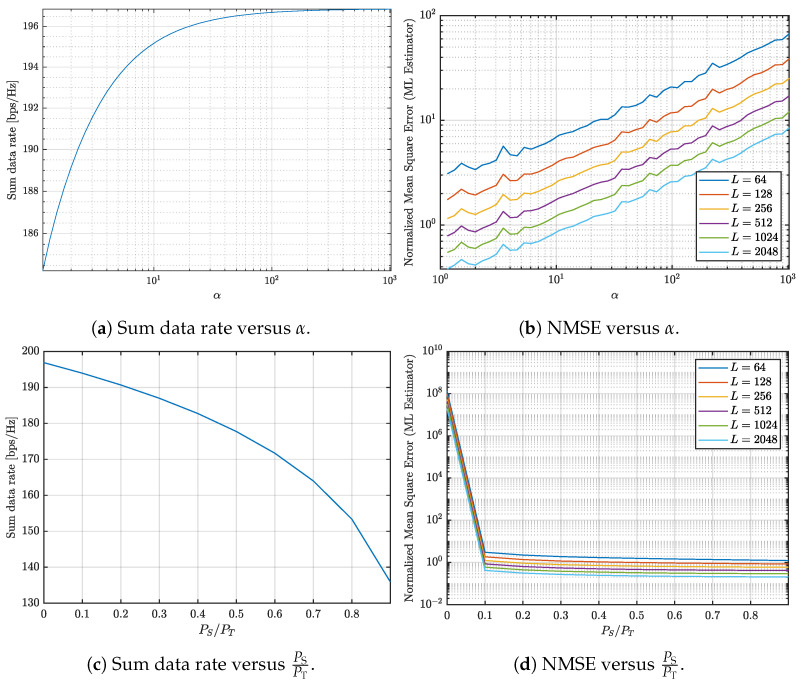
Analysis of the communication and sensing trade-off considering 5 UEs.

**Figure 2:**
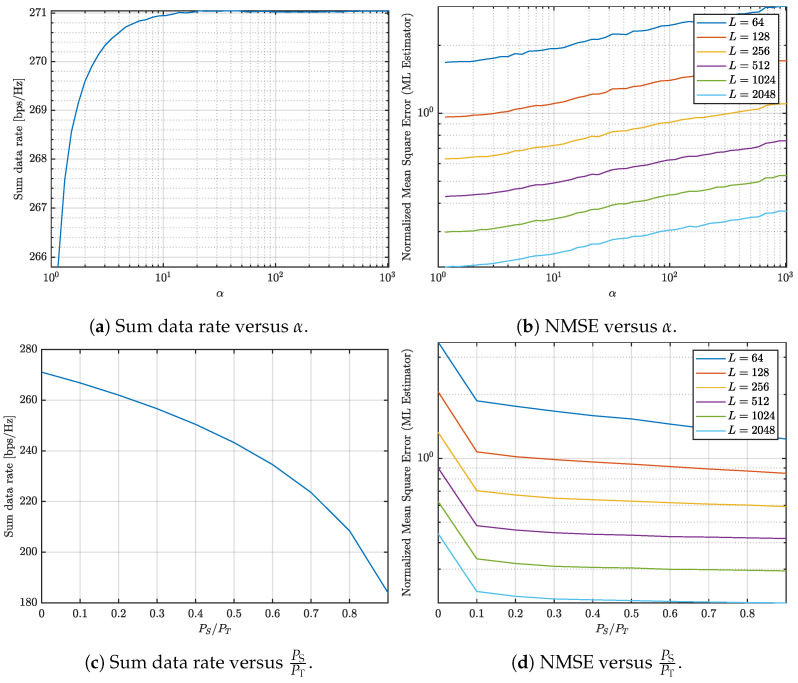
Analysis of the communication and sensing trade-off considering 8 UEs.

**Figure 3:**
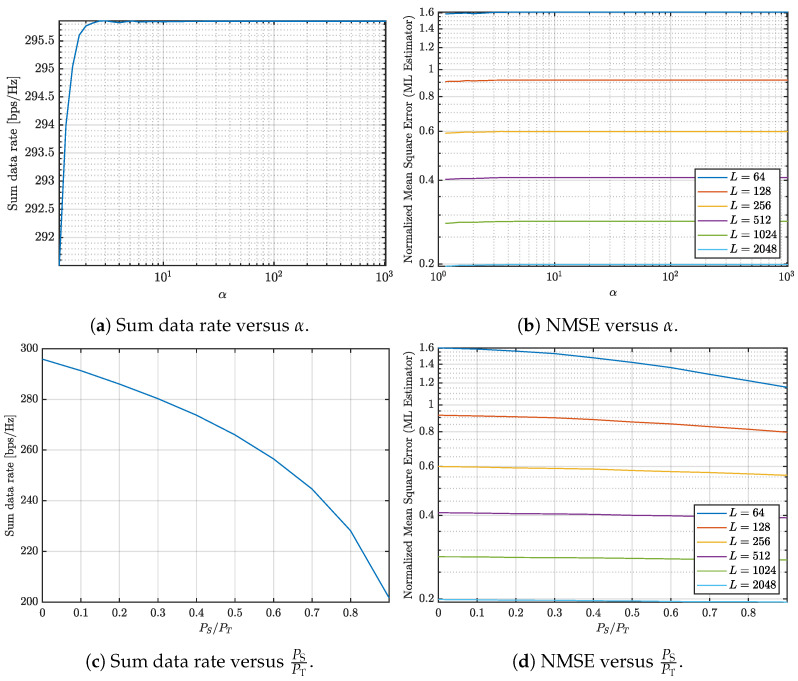
Analysis of the communication and sensing trade-off considering 10 UEs.

**Figure 4:**
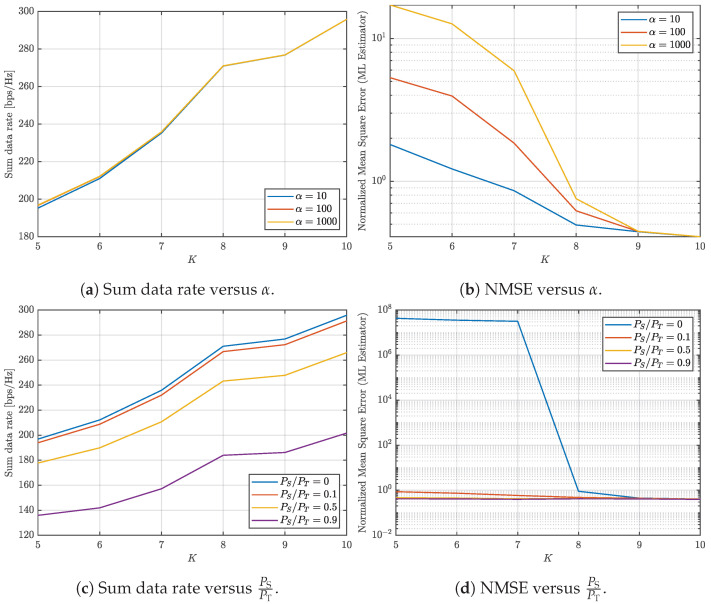
Analysis of the communication and sensing trade-off considering varying the number of UEs.

This paper investigates the trade-offs between communication and sensing in multi-user ISAC systems with varying numbers of UEs. The study jointly evaluates the achievable communication rate and the NMSE of the sensing channel estimation under different system configurations and design parameters, as illustrated in Figure 1, Figure 2, Figure 3 and Figure 4. Three representative scenarios are considered: (i) an underdetermined system with 5 UEs, (ii) a balanced configuration with 8 UEs, where the numbers of transmit and receive antennas are equal, and (iii) an overdetermined setup with 10 UEs, in which the number of receive antennas exceeds the number of transmit antennas.

In all cases, two parameters are varied to study their impact on the communication–sensing trade-off: the condition number threshold α and the fraction of transmit power allocated to sensing. For Figure 1, Figure 2 and Figure 3, α is varied from $10^{0}$ to $10^{3}$ in logarithmic steps of 0.1, while the sensing power fraction is swept from 0 to 0.9 in steps of 0.1. These ranges allow us to capture both extreme operating points: α=1 corresponds to the optimal sensing scenario, and a sensing power fraction of 0 corresponds to the optimal communication scenario. The intermediate values illustrate how varying the covariance conditioning and sensing power fraction jointly affects the trade-off between sum data rate and sensing NMSE. For Figure 4, representative thresholds α∈{10,100,1000} and sensing power fractions {0,0.1,0.5,0.9} are considered to highlight distinct operating regimes and characterize the continuous transition between communication- and sensing-oriented designs.

Figure 1 analyzes the communication and sensing trade-off when 5 UEs are allocated. Figure 1a illustrates the relationship between the sum data rate and the condition number. As the condition number increases, the sum data rate also rises. This behavior is expected, as a higher condition number implies a softer constraint on the sum of the covariance matrices, allowing greater flexibility in power allocation to maximize the sum data rate. However, the sum data rate eventually flattens at approximately 196 bps/Hz.

Figure 1b shows the NMSE as a function of the condition number for various OFDM symbol counts (L=64,128,256,512,1024,2048). The NMSE increases with the condition number, reflecting reduced accuracy in sensing channel estimation due to poor conditioning of the transmit covariance matrices. However, the NMSE decreases as the number of OFDM symbols (*L*) increases. This improvement occurs because a larger *L* provides more observations over time, enabling better averaging of noise.

Figure 1c demonstrates the sum data rate decline as more power is allocated to sensing. As the sensing power fraction ($P_{S}/P_{T}$) increases, less power remains available for communication, leading to a reduction in system capacity. Figure 1d illustrates the NMSE as a function of $P_{S}/P_{T}$ for different OFDM symbol counts. Introducing a dedicated sensing signal significantly improves the NMSE, particularly when a small fraction of power is allocated to sensing. Beyond this point, further increases in sensing power yield diminishing returns, as the NMSE stabilizes.

Increasing the condition number results in higher sum data rates and NMSE. Increasing the number of OFDM symbols enhances sensing accuracy because the larger symbol set improves the channel estimation process by mitigating noise through averaging. However, increasing the number of OFDM symbols requires larger delays on sensing channel estimation, and the channel has to be stable when transmitting those symbols. Therefore, increasing the number of OFDM symbols might not always be an option. Conversely, allocating additional power to sensing reduces overall system capacity. For systems where the number of transmit antennas exceeds the number of receive antennas, dedicating a small portion of power to sensing achieves an optimal balance. For example, allocating 10% of the total power to sensing decreases the sum data rate by only 3 bps/Hz, while dramatically reducing the NMSE from $1.9\times 10^{7}$ to 0.41. Achieving comparable accuracy without a dedicated sensing signal would result in a much larger loss in sum data rate, exceeding 10 bps/Hz.

After analyzing the trade-off behavior in a scenario where there are more transmit antennas (32 at the BS) than receive antennas (20 in total, with 4 antennas per UE across 5 UEs), it is interesting to explore what happens when the number of antennas is balanced between transmission and reception. Specifically, we consider a case with 32 antennas at the BS and 8 UEs, each equipped with 4 antennas, resulting in an equal number of transmit and receive antennas. Figure 2 examines the communication and sensing trade-off for this scenario.

In Figure 2a, the sum data rate is shown as a function of the condition number. As in the previous scenario, the sum data rate increases with the condition number, but the growth rate is slower compared to the case with 5 UEs. This difference arises because the system now has an equal number of transmit and receive antennas, leading to better conditioning of the sum of the signal covariance matrices. Similarly, Figure 2b depicts the NMSE as a function of the condition number. Here, the NMSE also increases more gradually than in the 5 UEs case. While increasing the number of OFDM symbols continues to improve sensing performance, the improvement in NMSE is less pronounced due to the more balanced antenna configuration.

Figure 2c highlights how increasing the sensing power fraction ($P_{S}/P_{T}$) reduces the sum data rate, consistent with the 5 UEs case. However, Figure 2d shows that the corresponding reduction in NMSE is less significant than before. This indicates that allocating power to sensing in a balanced system configuration is less impactful, as the system is inherently better conditioned for communication and sensing tasks.

Keeping the condition number below 10 achieves similar NMSE performance compared with having a sensing dedicated signal and has minimal impact on communication capacity. Therefore, limiting the condition number proves to be a more efficient strategy for maximizing the sum data rate and keeping a low NMSE compared to allocating additional power to sensing.

Finally, we analyze a scenario where the number of receive antennas exceeds the number of transmit antennas. Specifically, we assess a system with 10 UEs, corresponding to 32 transmit antennas at the BS and 40 receive antennas across the UEs. Figure 3a shows that the sum data rate remains unaffected, mainly as the condition number increases. This stability arises because the sum of the covariance matrices in this configuration is inherently well-conditioned, enabling the system to achieve near-optimal performance without requiring additional adjustments.

Similarly, Figure 3b illustrates that the NMSE remains relatively stable as the condition number increases, further confirming that matrix conditioning is less critical in this scenario compared to the previous cases.

Figure 3c demonstrates that allocating power to sensing significantly reduces the sum data rate. However, Figure 3d shows that the corresponding improvement in NMSE is minimal. This indicates that dedicating power to sensing in this configuration is not worthwhile, as the marginal benefits in sensing accuracy do not justify the substantial loss in communication capacity.

To complement the previous analysis, we present additional graphs illustrating how the system transitions from underdetermined to overdetermined as the number of users increases. To analyze this transition, we consider scenarios with 5 to 10 users, three condition numbers (α) values (10, 100, and 1000), and four power allocation ratios ($P_{S}/P_{T}$): 0, 0.1, 0.5, and 0.9. Figure 4 presents the results for the sum data rate and NMSE across different numbers of UEs, condition numbers, and power allocation ratios.

Figure 4a displays the sum data rate as a function of the number of users for various condition number values. The results show an increasing trend in the sum data rate, but the growth slows noticeably after 8 users. This behavior reflects the transition from an underdetermined system (fewer than 8 users, where the DoF exceed the number of active receivers, leading to underutilization) to an overdetermined system (8 or more users, where the number of active receivers matches or exceeds the available DoF). In the overdetermined regime, adding more users diminishes gains in the sum data rate, as the system’s resources are already fully engaged.

Regarding the NMSE, Figure 4b demonstrates that limiting the condition number reduces the error. However, after 9 users, the system becomes inherently well-conditioned due to the overdetermined configuration, eliminating the need for further constraints. This indicates that the presence of more users naturally balances the system, improving performance.

Next, we analyze Figure 4c,d to evaluate the impact of allocating power to sensing. In Figure 4c, the sum data rate increases as users grow but decreases with higher $P_{S}/P_{T}$. These results are expected, as dedicating more power to sensing reduces the power available for communication. However, Figure 4d highlights that using a dedicated sensing signal significantly reduces the NMSE, particularly when the system is underdetermined (fewer than 8 users). In this regime, allocating just 10% of the total power to sensing is sufficient to achieve acceptable error levels. Beyond this point, further increases in sensing power offer minimal improvements in error reduction but result in a significant degradation of the sum data rate.

## 6. Conclusions

This work examined the trade-offs between communication and sensing in wireless ISAC systems, focusing on the effects of user load, transmit covariance conditioning, and sensing power allocation. Through systematic analysis, it was shown that in the underdetermined regime (few users), allocating a small fraction of transmit power to sensing can substantially improve the sensing NMSE with only a limited reduction in achievable data rate. As the system transitions toward the overdetermined regime, where the number of receive antennas exceeds the number of transmit antennas, additional users bring diminishing performance gains. In this case, the system naturally exhibits favorable conditioning, and further restriction of the condition number or increase in sensing power allocation yields minimal benefit. Overall, the results indicate that sensing-oriented power allocation is most advantageous under low-user-load conditions, while performance in highly loaded or overdetermined systems tends to stabilize without additional intervention. These insights motivate the development of adaptive allocation and conditioning strategies that dynamically respond to network load and environmental factors to maintain efficient joint operation.

Future research may explore the integration of the proposed approach into multi-carrier systems and time-varying, dynamic channel environments, where adaptive tuning of sensing and communication constraints can support real-time reconfiguration and robust ISAC operation. In this context, incorporating reconfigurable intelligent surface (RIS) could provide additional channel degrees of freedom, enhancing both communication throughput and sensing accuracy. By carefully designing RIS reflection patterns, the system can improve spatial diversity, channel conditioning, and the flexibility of trade-offs between data rate and sensing performance. Furthermore, learning-based control of the condition number constraint, combined with joint optimization of RIS configurations and mobility- or energy-aware strategies, represents a promising direction for improving scalability and enabling practical deployment in next-generation wireless networks.

## Figures and Tables

**Table 1:** Evaluation configuration.

Simulation parameters	
Scenario	UMa
Number of BS	1
Number of UE *K*	5–10 (varied)
Number of target	1
Number of seeds	10,000
Number of OFDM symbols to estimate the channel *L*	{64,128,256,512,1024,2048}
Condition-number thresholds α	$\left\{10^{0.1:0.1:3}\right\}$
Sensing power ratio $P_{S}/P_{T}$	{0:0.1:0.9}
Baseline evaluation configuration parameters	
Carrier frequency for evaluation	10 GHz
Sampling frequency	300.72 MHz
Total Bandwidth	100 MHz
Number of subcarriers	1
BS antenna height	25 m
Total transmit power $P_{T}$	100 W
Additional parameters for system-level simulation	
Number of BS antenna elements ($N_{t}$)	32
Number of UE antenna elements ($N_{u}$)	4
UE height	1.5 m
Target height	1.5 m

## Data Availability

The data presented in this study are available on request from the corresponding author.
